# SELF-RATED HEATH PREDICTS INCIDENT MODERATE/SEVERE DISABILITY IN AGING MEN: THE MANITOBA FOLLOW-UP STUDY

**DOI:** 10.1093/geroni/igae098.2403

**Published:** 2024-12-31

**Authors:** Phil St John, Scott Nowicki, Robert Tate

**Affiliations:** University of Manitoba, Winnipeg, Manitoba, Canada; University of Manitoba, Winnipeg, Manitoba, Canada; University of Manitoba, Winnipeg, Manitoba, Canada

## Abstract

**Background:**

There is evidence that Self-Rated Health (SRH) predicts death, but less evidence that SRH predicts new disability. Objective: To determine if SRH predicts moderate/severe disability in a prospective cohort study of aging men.

**Methods:**

We conducted an analysis of a prospective cohort study of men who qualified for air crew training in the Royal Canadian Air Force, which began in 1948. Since 2004, an annual survey is sent out which measures age-reference SRH, functional status, and the Short-Form-36 (SF-36). In 2004, 796 men were alive, responded to the questionnaire and did not have disability. Functional status has been measured with an annual questionnaire which includes items on Basic Activities of Daily Living (BADLs) and Instrumental ADLs (IADLs). We considered impairment to be two or more BADLs impaired (of five considered) or three or more impaired IADLs (of seven). We calculated the median time to disability, and compared groups with a log rank test. We then constructed a proportional hazards model for the outcome of new disability.

**Results:**

SRH was associated with incident disability: Compared to those with Excellent SRH, the Hazard Ratio (95% confidence interval) was 1.79 (1.19, 2.68) for those with Very Good SRH; 2.87 (1.91, 4.30) for those with Good SRH; and 2.40 (2.04, 5.65) for those with Fair/Poor/Bad SRH. This association persisted after adjusting for age, the SF-36 and chronic diseases. Conclusions: SRH predicts new disability.

